# Estimating Landholders’ Probability of Participating in a Stewardship Program, and the Implications for Spatial Conservation Priorities

**DOI:** 10.1371/journal.pone.0097941

**Published:** 2014-06-03

**Authors:** Vanessa M. Adams, Robert L. Pressey, Natalie Stoeckl

**Affiliations:** 1 Australian Research Council Centre of Excellence for Coral Reef Studies, James Cook University, Townsville, Queensland, Australia; 2 Research Institute for the Environment and Livelihoods and Northern Australia National Environmental Research Program Hub, Charles Darwin University, Darwin, Northern Territory, Australia; 3 School of Business and Cairns Institute, James Cook University, Townsville, Queensland, Australia; University of Kent, United Kingdom

## Abstract

The need to integrate social and economic factors into conservation planning has become a focus of academic discussions and has important practical implications for the implementation of conservation areas, both private and public. We conducted a survey in the Daly Catchment, Northern Territory, to inform the design and implementation of a stewardship payment program. We used a choice model to estimate the likely level of participation in two legal arrangements - conservation covenants and management agreements - based on payment level and proportion of properties required to be managed. We then spatially predicted landholders’ probability of participating at the resolution of individual properties and incorporated these predictions into conservation planning software to examine the potential for the stewardship program to meet conservation objectives. We found that the properties that were least costly, per unit area, to manage were also the least likely to participate. This highlights a tension between planning for a cost-effective program and planning for a program that targets properties with the highest probability of participation.

## Introduction

Private land conservation is becoming more prominent and important as expansion of strict protected areas is increasingly constrained by reduced availability of land, insufficient budgets for acquisition, and escalating management costs of small, isolated reserves [1]–[3]. Longstanding conservation programs on private land include the US Conservation Reserve Program [4] and, in Australia, the Victorian Bush Tender Program [5].

Farmers, Indigenous owners and other private landholders manage approximately 77% of Australia’s land area. This statistic alone indicates that conservation on private land is integral to Australia’s biodiversity conservation strategy [6]. All Australian states and territories have legislation for conservation covenanting on private properties, although some state programs are longer established and cover larger areas than others [7]. Several states have competitive tendering for conservation contracts including the Victorian Bush Tender Program [5], the New South Wales Environmental Services Scheme, and the Queensland Nature Assist program.

Understanding landholders’ willingness to participate has two important implications for private land conservation. First, this understanding will shape policy for the design of incentives. For example, factors specific to program design, such as proposed land management, constraints on land title, and delivery of incentives, will influence willingness to participate [8]. Typical approaches to assess the design and viability of stewardship programs include methods such as choice modelling and auctions [9], [10]. While these approaches will reveal expected participation levels and provide insights into the effective design of programs, they are not typically structured to assess whether a program is likely to achieve spatial conservation objectives.

The second implication of information on willingness to participate is that identifying willing landholders is vital to identifying areas that are both valuable for achieving objectives and feasible for conservation action. For example, a map of landholders’ willingness can be used to design a configuration of protected areas that will, at least in theory, be more easily implemented because it selects those properties owned or managed by people more likely to engage in formal protection [11]. Alternatively, a map of landholders’ willingness can be used to assess the likely spatial configuration of voluntary, private protected-area management resulting from a conservation auction, demonstrating the scope for an auction program to achieve conservation outcomes.

Combining willingness and spatial conservation priorities will allow for conservation programs to enhance the likelihood that spatial conservation objectives are met. One example of the potential to incorporate spatial conservation priorities into the auction process is the Western Australian Conservation Auction, in which assessment of the benefits offered by properties accounted for complementarity of conservation values between bids [12]. This process demonstrated the potential to integrate well-developed auction processes with spatial planning to establish sets of private conservation areas that maximized the achievement of conservation objectives within budgets. The conservation outcomes of a program might also depend on aspects of spatial configuration of the properties selected [13]–[15], for example to achieve objectives related to connectivity and buffering from surrounding land uses. The potential to consider configuration as well as representation of ecosystems and species has been demonstrated in applications to protect and restore private lands [16], [17]. Furthermore, configuration of properties might have important social implications in addition to ecological benefits. For example, landholders might be more inclined to participate in a program if their neighbours are participating, one reason being the added certainty that benefits associated with improved management would not be at risk from unmanaged threats nearby (e.g. spread of unmanaged weeds or fire) [18], [19].

Of the Australian states and territories, the Northern Territory’s policies and funding for conservation on private lands are the least developed, with financial support for conservation covenants and management agreements under consideration. Therefore, we undertook a pilot study in the Daly Catchment to assess the potential for such programs to meet conservation objectives. The program under consideration is for stewardship payments to leverage already extensive routine land management by altering or extending land management practices to meet conservation objectives on private lands. The program would include covenants, which are perpetual titles on private land, as well as management agreements, which are legal agreements between the Government and landholders. We have examined aspects of designing the program such as costs and payment structures [18]. Here, we report on landholders’ willingness to participate in such a program.

Our study had three aims. The first was to assess landholders’ willingness to participate and inform the design of a stewardship-payment program in the Daly Catchment, Northern Territory. We used a choice experiment to estimate the probability of participation in the program relative to: 1. contract type (covenant versus management agreement); 2. payment amount; and 3. required change in proportion of property managed for conservation. These factors have been identified as important in influencing participation in programs in other regions (e.g., participants relying on production for income may require higher levels of compensation) [20], [21]. Choice modelling can estimate the effects of combinations of factors on participants’ choices and is therefore useful for designing policies [22], [23] and has been used in other regions to explore the influence of attributes such as compensation and duration of contract on willingness to participate [21], [24]. The choice model allowed us to estimate the expected level of participation in a program in the Daly, which can indicate the viability of the program more broadly and provide guidelines to the Government about adequate budgets to meet desired participation levels. The choice model also allowed us to examine landholders’ preferences for the two mechanisms presented (covenants and management agreements) and how these preferences varied with respect to payment amount and required change in proportion of property to be managed for conservation.

Our second aim was to assess the potential of the stewardship program to meet spatial conservation objectives. We therefore predicted spatially landholders’ willingness to participate at the resolution of individual properties and incorporated these predictions into conservation planning software. Predicting willingness to participate for individual properties allowed us to consider the potential spatial distribution of participating properties and therefore the likely conservation outcomes relative to vegetation types mapped across the catchment. Understanding whether a stewardship program would have the desired impacts of achieving adequate protection for spatially variable conservation features is an important step in scoping a program that has been underutilized.

The third aim of our study was to analyze how the interactions between willingness to participate and conservation costs can influence solutions identified in spatial conservation planning. The potential correlations between conservation costs and willingness to participate have not yet been examined, although they could determine the success of a conservation program. For example, if costs and willingness are negatively correlated then an incentive program would probably be feasible: the properties most likely to be included would also be the most cost-efficient to engage. However, a positive correlation would mean that the most willing landholders also have the least cost-efficient properties, posing difficulties for the design of an incentive scheme. We examined the implications of interactions between costs and willingness to participate in our study region. Our study is the first to incorporate both spatially variable willingness to participate and spatially variable costs. Therefore, this is the first study to elucidate how these two components of the planning problem interact and potentially enhance or constrain capacity to meet conservation objectives.

## Materials and Methods

### Ethics Statement

This study was approved by James Cook University’s Human Ethics Committee (H3283).

### Study Area

**Figure 1:**
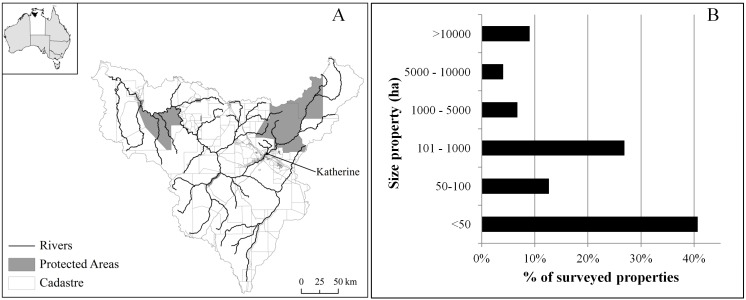
The Daly catchment and pastoral and horticultural properties. The map inset shows the Northern Territory with pale shading and the Daly catchment in black. A) Rivers, protected areas and boundaries of properties (cadastre). Two large national parks extend into the north-east corner of the catchment: Nitmiluk National Park and the southern portion of Kakadu National Park. Fish River Indigenous Protected Area is in the north-west portion of the catchment. B) Size distribution of the 440 properties included in our survey.

The study area was the whole of the Daly River catchment in the Northern Territory, covering approximately 5.2 million ha and extending from the coastline south-west of Darwin to 250 km inland (Figure 1A). The Daly River is one of the major river systems in the Top End. Riparian strips in the Daly catchment contain some of the most extensive gallery (rainforest) vegetation in the Northern Territory. Five of the sixty-seven sites of conservation significance identified in the Northern Territory occur in the Daly Catchment [25]. Approximately 10% of the catchment is protected by national parks, such as Nitmiluk Gorge, and Indigenous Protected Areas, such as Fish River. However, protection is not representative across the 105 mapped vegetation types, with 48 having at least 10% area protected and the remaining 57 having less than 10% area protected. Therefore, considerable effort is still needed to ensure adequate and representative protection of the vegetation types in the Daly catchment. In addition, the Daly catchment area is regarded as a highly prospective region for further development. The potential for future pressure to clear native vegetation makes the area a high priority for conservation to ensure valued areas are adequately protected.

The many conservation priorities in the catchment are unlikely to be addressed with further acquisition for national parks because of the large property sizes and correspondingly large acquisition and management costs. Instead, the region is suitable for off-reserve programs involving stewardship payments in conjunction with conservation agreements between the Government and landholders. The mean size of private properties in the Daly is ∼10,500 ha (median size 90 ha). Properties larger than 5,000 ha represent approximately 13% of landholders but about 90% of the catchment’s private land (Figure 1B). Therefore, engaging with relatively few landholders has the potential for extensive conservation benefits. In addition to engaging with private landholders, the Government is interested in funding new Indigenous Protected Areas. These are agreements between traditional owners and the Australian Government, considered to be similar to national parks. Funding both Indigenous Protected Areas and a stewardship program on non-Indigenous properties would equitably provide opportunities for all Daly residents to access financial support for conservation management.

### Discussions with the Northern Territory Government

The Northern Territory is the only Australian jurisdiction without well-established arrangements for covenants and conservation management agreements. Therefore, we used the structure of the Queensland Nature Refuge program, which supports establishment of covenants on freehold and leasehold land, as the basis for designing our survey questions. The state of Queensland has more private land under covenant (referred to as Nature Refuges) than any other Australian jurisdiction [26] and has recently implemented legislation, called the Delbessie Agreement, to encourage participation in the program by landholders on extensive leasehold properties [27]. Under the Delbessie Agreement, lessees with properties identified as having conservation value must either enter into a Nature Refuge agreement and be rewarded with a 10-year lease extension or elect to have their properties acquired if they do not wish to participate.

We undertook a series of conversations with the Northern Territory Government that indicated that the relevant agency would consider a scheme similar to Queensland’s Nature Refuge program and that the Daly catchment was a high priority area for trialling such a program. We therefore designed our survey with the assumptions that the Northern Territory would model its covenant program for private land on Queensland’s and that legislation similar to Queensland’s Delbessie Agreement would be considered to support the environmentally sustainable, productive use of rural leasehold land.

Based on a pilot survey, below, and discussions with the Northern Territory Government, including staff working on private protected-area initiatives and spatial conservation planning, we identified three realistic parameters of a stewardship program. First, the Government would pay a premium to engage landholders in conservation covenants in preference to management agreements because of the perceived benefits of permanent title for conservation (payments of 150% of actual stewardship costs for covenants as opposed to 100% of actual stewardship costs for management agreements). Second, most landholders are currently not managing any areas for conservation, over and above routine property management. Third, landholders participating in the stewardship program would be required to manage several small patches on their property for conservation.

Officers of the Northern Territory Government also indicated that they would consider equal funding for Indigenous Protected Areas alongside funding for a stewardship program on private land to ensure that funds were available for conservation across tenures.

### Choice Modelling Experiment and Survey Methods

The survey included questions about the characteristics of landholders and properties, current expenditures on land management and conservation management, and other information specific to the choice experiments. For the choice experiment, respondents were asked to consider the hypothetical scenario of a stewardship program with three alternatives for landholders: conservation covenant, conservation management agreement, or sell property. Choice experiments typically include a status-quo or default option. In our design, we did not include an ‘opt-out’ option because we wanted to mirror legislation similar to the Delbessie Agreement. Under that arrangement, ‘sell property’ could be considered the opt-out or status quo because it is the only option for landholders unwilling to place portions of their properties under covenant. Not all on-farm conservation programs have similar ‘conserve or sell’ clauses, so the results of this experiment are not transferrable to those situations. Indeed, the probabilities of participation estimated here will likely exceed those obtained in situations where neither sale nor participation is necessary. Our results are therefore optimistic estimates of environmental outcomes from a stewardship program and we would expect larger shortfalls in meeting conservation objectives in situations with the default option of not participating.

Based on landholders’ attitudes and responses to the Nature Refuge program, we hypothesized that willingness to participate in a program would depend on the type of agreement (covenant or management agreement), the proportion of property already set aside for conservation, the additional proportion of property to be set aside for the program, and the financial payment relative to costs of conservation management above day-to-day land management costs [8], [20]. Adams et al. [18] estimated the additional costs of conservation management above day-to-day land management costs for landholders in the Daly and we term these costs ‘stewardship costs’. We assumed for our study that landholders would receive stewardship payments as a function of their additional costs to achieve conservation objectives.

**Figure 2:**
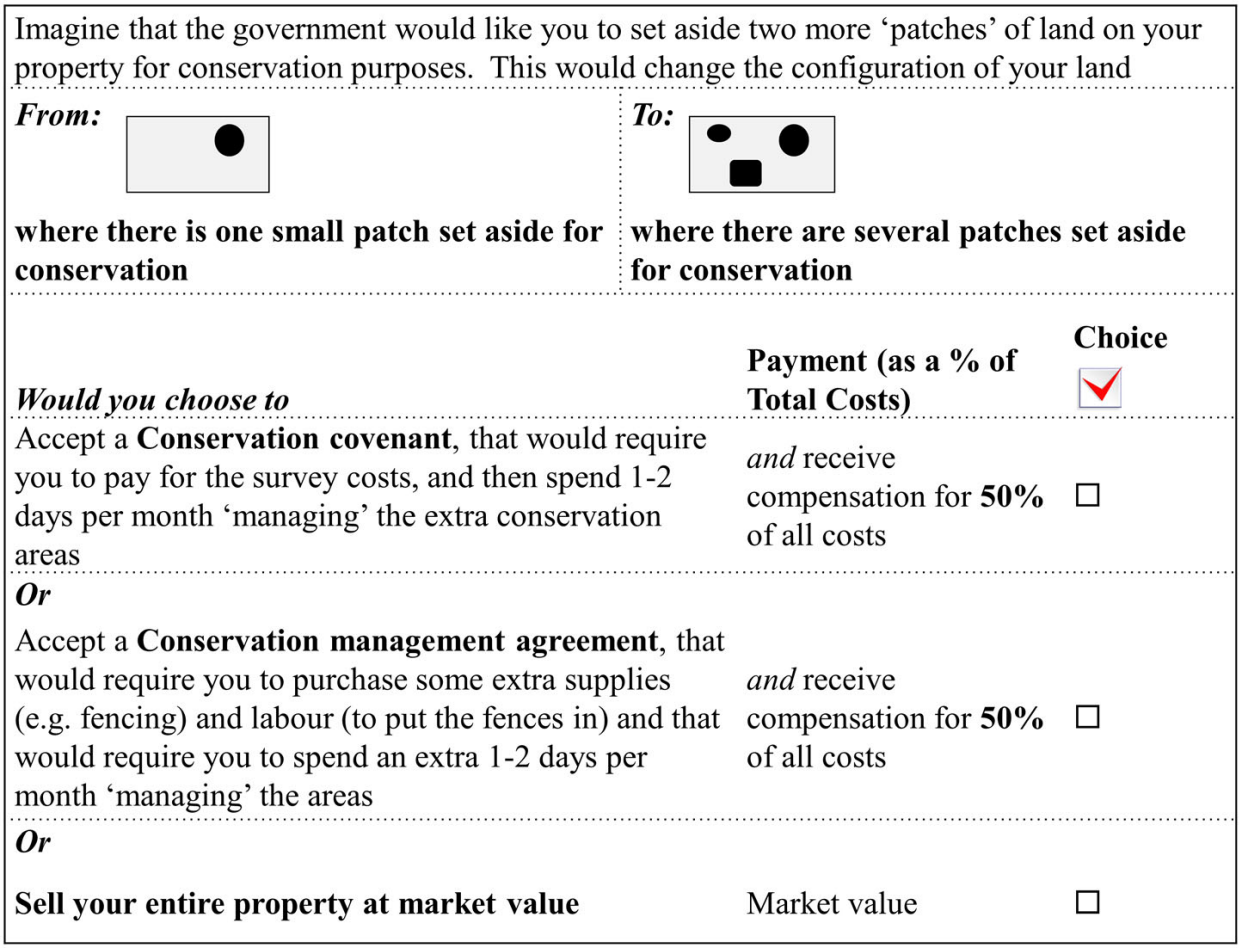
Example choice set presented to respondents in survey.

**Figure 3:**
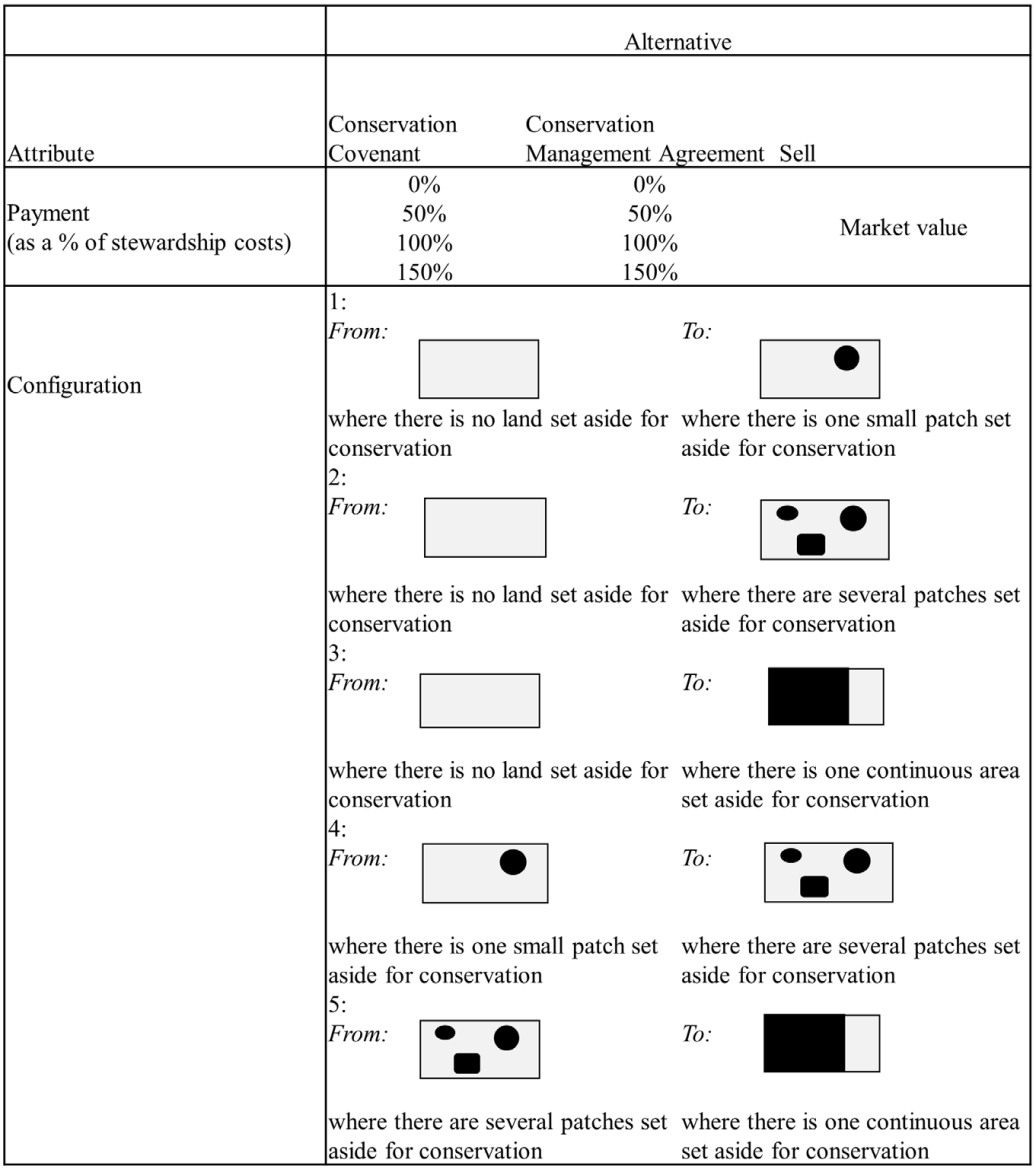
Attribute levels and changes for the choice experiment. The survey provided respondents with three alternatives: conservation covenant, conservation management agreement, or sell property. The choice experiment explored two attributes that might influence respondents’ choices: payment level as a percentage of stewardship costs (defined here as the additional costs of managing land for conservation, over and above routine property management) and change in extent and configuration of conservation management, defined relative to current configuration (*From*) and future configuration (*To*). We considered four payment levels and five changes in configuration.

In a pilot study, we tested different attributes of a stewardship program to ensure they were cognitively accessible to respondents. Based on the pilot study, we represented financial payment as a percentage of stewardship costs because these costs will vary with current management activities and characteristics of properties, including size. We assumed that financial payments would range from 0% to 150% of stewardship costs (presented to survey respondents as a payment relative to actual costs incurred, Figure 2), and used incremental amounts across that range to allow interpolation between points in our model (Figure 3). We represented the required change in proportion of property set aside for conservation with five representative combinations identified from the pilot study (Figure 3). We constructed the choice sets using a full factorial design, resulting in 80 different combinations (4 covenant payments×4 management agreement payments×5 changes in proportion of property set aside). Because 80 choice sets would be too demanding for a respondent, we chose a blocked full factorial design; blocking is a common way to handle the trade-off between maximising the data collected from each respondent and fatigue of the respondent [28]. The choice sets were blocked into 8 versions of the choice experiment. Each participant was randomly assigned a block of ten choice sets and we ensured that the received responses were evenly distributed across the 80 choice sets. Respondents were given a set of definitions for alternative stewardship arrangements or sale of property using an information box and then asked to choose the preferred option in each choice set (example in Figure 2).

For our survey, we considered only land parcels in the catchment of 10 ha or larger and excluded properties within the town of Katherine (Figure 1). Properties in the town or smaller than 10 ha are probably not good candidates for conservation agreements because they are predominantly residential and unmanaged. We sent surveys to all landholders eligible for private land stewardship agreements, defined here as all 440 pastoralist landholders [see 18 for more details]. We used the Dillman tailored design method [29]. Of the 440 landholders contacted, 25 requested to be removed from the survey and 50 addresses were no longer active, leaving a total of 365 possible respondents. The response rate to the survey (about 25%, or 92 of 365 landholders, with 710 choice sets completed) was in line with similar surveys in the region [30], [31]. Responses were also representative of property size and types across the catchment [18] (Survey S1). Based on Orme’s rule of thumb [32], [33] the target number of respondents was 125. While our achieved response (92) was approximately 25% less than the target and we acknowledge that a small sample is likely to be high risk, our estimated coefficients were all statistically significant suggesting that our sample size was adequate.

### Choice Experiment Analysis

We analyzed the choice sets using a conditional mixed-effects logit model in STATA version 9. Based on the choice experiment, the probability of an individual *i* choosing an alternative *m* is given by
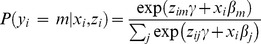
where alternative specific variables for individual *i* for alternative *m* are given by *z_im_* and coefficients are denoted by *γ*, case-specific variables for individual *i* are given by *x_i_*, and coefficients are denoted by *β*. In our choice experiment, conservation payments were alternative-specific while conservation configuration was case-specific and landholder-specific variables were included as case-specific variables. We explored a range of landholder-specific variables including size of property, engagement in conservation efforts, land use, number of years on property, and natural characteristics of properties [18]. In our final model we included the only two statistically significant landholder-specific variables: size of property (*ln(property size, ha)*); and a binary flag indicating whether the landholder was currently engaged in conservation management (*conservation flag*) (Table 1). Ideally, we would have also tested whether sale values of properties influenced landholders’ choices to sell, but reliable sales data were not available for the region.

**Table 1:** Conditional mixed-effects logit model.

Variable	Coefficient		SE
CC intercept	0.3704		0.3861
CMA intercept	0.3788		0.3492
Payment	0.0133	***	0.0012
Configuration 2, CC	−1.1400	***	0.3420
Configuration 3, CC	−1.3841	***	0.3562
Configuration 4, CC	−1.0396	**	0.3442
Configuration 5, CC	−1.1116	***	0.3336
Configuration 2, CMA	−0.6737	**	0.3123
Configuration 3, CMA	−1.0710	***	0.3216
Configuration 4, CMA	−0.4958	*	0.3091
Configuration 5, CMA	−0.8896	**	0.3162
Conservation flag, CC	2.2508	***	0.2935
Conservation flag, CMA	1.3770	***	0.2625
ln(property size), CC	−0.6335	***	0.1230
ln(property size), CMA	−0.4577	***	0.1004
N (Choice sets)	710		
Log L	−654.32		
rho^2^	0.16		

CC indicates conservation covenant; CMA indicates conservation management agreement. Configuration was coded as a set of dummy variables (corresponding to alternative changes in configuration in Figure 2) with configuration 1 chosen as the status quo.

*p<0.05,

**p<0.005,

***p<0.001.

### Application of Choice Model

We used our final choice model (Table 1) for two purposes. First, we explored how the probability of participation was affected by different payment levels, to understand how to maximize participation. Using the survey sample averages, we estimated the catchment-wide average probability of participation in covenants and management agreements based on three payment scenarios: 50% of stewardship costs for both conservation covenants and conservation management agreements; 100% of stewardship costs for both conservation covenants and conservation management agreements; and 150% of stewardship costs for conservation covenants and 100% of stewardship costs for conservation management agreements. For these scenarios, we assumed configuration 2 (no patches currently set aside for conservation and landholders would be required to set aside several small patches for conservation in the future). This was the most likely configuration across the properties in the catchment. These scenarios reflect discussions held with the NT Government about the likely design of the payment program (see section on discussions, above, for further detail).

The second use of the choice model was to create a map of expected probability of participation of individual properties so that our planning scenarios could preferentially select properties with higher probabilities of participation. For each property, we therefore estimated probability of participation assuming payments of 150% for covenants and 100% for management agreements and configuration 2 (no patches currently set aside for conservation and several patches to be set aside for conservation in the future).

### Spatial Planning Using Marxan with Zones

We conducted a spatial planning exercise to demonstrate how a map of estimated probability of participation can be used to design configurations of properties that contribute to conservation objectives. In many settings, any one spatial configuration identified during a planning process will change dynamically as planners engage with stakeholders and the actual, as opposed to estimated, willingness of landholders to participate is revealed [34]. If landholders identified in the initial configuration refuse to participate, the configuration would be iteratively updated until the full budget was exhausted. For our study, we assumed that the identified configuration of properties would be used to direct first engagement with landholders, so we compared initial configurations between several scenarios (Table 2 and below).

**Table 2:** Scenarios compared using Marxan with Zones.

Scenario	Zones included (proportionalcontribution of zones toobjectives in parentheses)	Cost IPA	Cost stewardship
Scenario 1 - Uniform costs	1 – National Park (1)	Area	Area
	2 – IPA (1)		
	3 – Stewardship (1)		
	4 – Never Clear (0.7)^a^		
	5 – Available (0)^b^		
Scenario 2 - Variable costs	As above	$2.25 per ha	Estimated expected stewardship costs per ha
Scenario 3 - Uniform costs + probability ofparticipation	As above	Area	Area
Scenario 4 - Variable costs + probability ofparticipation	As above	$2.25 per ha	Estimated expected stewardship costs per ha

We defined scenarios in terms of zones considered, proportional contribution of zones to conservation objectives, costs of management in Indigenous Protected Areas (IPAs), and costs of stewardship (private pastoral zone).

a Areas covered by legislation that prevents clearing, assuming that this legislation is fully effective for ensuring that the area will not be cleared but contributes less than a protected area managed for conservation. For example these areas may be grazed or have invasive weeds or feral animals present, which may result in lower biodiversity compared to conserved land [49].

b Currently not managed for conservation but available for management either with IPA or stewardship.

We chose to explore how conservation objectives would be met across the catchment, subject to constraints on funds and area dedicated to conservation, by both Indigenous Protected Areas and stewardship agreements collectively. Indigenous Protected Areas, although very different mechanisms from covenants and management agreements, were important to consider because of the Government’s preparedness to consider them as part of an overall approach to nature conservation. Using Marxan with Zones [35] we planned for five zones: 1. national parks; 2. Indigenous Protected Areas (IPAs); 3. stewardship agreements; 4. riparian buffer areas and other sites that are protected under clearing guidelines for the Daly catchment, termed here the ‘never clear’ zone; and 5. un-engaged areas used for production but not conservation management, termed here the ‘available’ zone. We chose these zones to account for existing formal conservation areas (zone 1) or parts of the catchment protected from clearing through other measures (zone 4) and to explicitly plan for new conservation areas through IPAs (zone 2) and stewardship agreements (zone 3). Because of the size of the optimization problem (large number of planning units, features, and zones), we chose to select properties for a generalized ‘stewardship agreement’ zone without differentiating between covenants and management agreements. For the planning process, we assumed a single time step in which areas were identified for engagement for stewardship or Indigenous Protected Areas and that engagement and conservation management would continue. It was beyond the scope of our study to predict the vagaries of iterative adjustments to configurations [34] as individual landholders are engaged and some decline participation.

Marxan, a widely used conservation planning tool, uses the simulated annealing algorithm to minimize the objective function score:
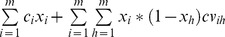
subject to the constraint that objectives are met:



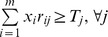



For *m* planning units, *n* features, *r_ij_* is the occurrence level of feature *j* in site *i* and *x_i_* is the control variable that indicates which planning unit is in, or out of, the reserve system. Marxan with Zones generalizes this approach by increasing the number of states or zones to which a planning unit can be assigned.

We used Marxan with Zones to examine, for four scenarios, possible spatial configurations of Indigenous Protected Areas and stewardship agreements. The scenarios (Table 2) were designed to examine the influence of variable costs and variable probability of participation on: 1. the spatial configuration of properties selected for a stewardship program; and 2. the capacity to meet conservation objectives within budget constraints.

#### Scenario 1 (uniform costs)

Cost of each planning unit is equal to its area; not considering probability of participation.

#### Scenario 2 (variable costs)

Cost of each planning unit is equal to the expected cost of participation in a stewardship program; not considering probability of participation.

#### Scenario 3 (uniform costs + probability of participation)

Cost of each planning unit is equal to its area; considering probability of participation.

#### Scenario 4 (variable costs + probability of participation)

Cost of each planning unit is equal to the expected cost of participation in a stewardship program; considering probability of participation.

We divided all properties in the catchment into planning units of square 25 ha grids (n = 212,173). Relatively small planning units allowed us to capture already protected areas in ‘never clear’ zones within properties and to identify spatial heterogeneity of conservation priority within properties (as opposed to identifying only whole properties as priorities). To control the aggregation of selected areas [35], we identified the zone boundary cost for each scenario with the method of Stewart & Possingham [36]. Because properties were divided into multiple planning units we checked that the percentage of each property selected for stewardship agreements was in line with the configuration assumptions made for calculating probability of participation. We included quantitative objectives for 105 vegetation types and the 5 sites of conservation significance in the Daly catchment, to give a total of 110 conservation features. The sites of conservation significance within the Daly River catchment have been assessed as either nationally or internationally significant and include features such as the Daly River, Anson Bay and Floodplains and Western Arnhem Plateau [25]. Based on discussions with the Northern Territory Government Department for Natural Resources, Environment, The Arts and Sport (NRETAS), our objectives were 30% of the current extent of each vegetation type (because pre-clearing data were not available) and 100% of each site of conservation significance. The Northern Territory has clearing guidelines for the Daly that allocate buffers around sensitive vegetation or other features that cannot be cleared (e.g. a required 250 meter buffer around all streams) [37]. Therefore, we locked all required buffers (the ‘never clear’ zone in Marxan with Zones) into the selected conservation configuration for all scenarios. In addition, we locked in all existing national parks and Indigenous Protected Areas. We assumed that the different zones contributed differentially to conservation objectives (Table 2), reflecting different levels of commitment of management to conservation.

Marxan with Zones minimizes the total cost of the zoning plan *C*:
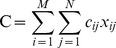
where *x_ij_* = 1 if the *i^th^* planning unit is included in the *j^th^* zone, subject to the constraint that a planning unit can only be placed in one zone. For scenarios 1 and 3, a uniform cost was used for planning units (i.e. cost was equal to the area of the planning unit). For scenarios 2 and 4 a spatially variable cost was used for planning units. For properties under consideration for Indigenous Protected Areas, we used an expected conservation management cost per ha of $2.25 [25]. Because we did not distinguish between covenants and management agreements in our optimization problem, we calculated an expected stewardship cost per property based on: 1. covenants receiving a premium over management agreements (150% of total costs as compared to 100% for management agreements); 2. the estimated probability of participation in each mechanism; and 3. the explanatory model of stewardship costs from Adams et al. [18] to estimate the per-ha costs of stewardship payments. Therefore, we calculated the total expected cost per ha of stewardship payments per property as:



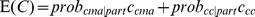
where *prob_cma_* is the probability of landholder *i* selecting a conservation management agreement calculated with the choice model, *prob_cc_* is the probability of landholder *i* selecting a conservation covenant calculated with the choice model, *prob_cma|part_* is the probability of landholder *i* selecting a conservation management agreement given the landholder has agreed to participate in the program (equal to *prob_cma_*/(1-*prob_sell_*)), *prob_cc\part_* is the probability of landholder *i* selecting a conservation covenant given the landholder has agreed to participate in the program (equal to *prob_cc_*/(1-*prob_sell_*)), *c_cma_* is the cost of stewardship payment to landholder *i* based on Adams et al [18], and *c_cc_* is 150% of *c_cma_*. We then calculated the management cost of each planning unit from the calculated per-ha expected cost of stewardship.

For scenarios 2 and 4 we ran Marxan with Zones to achieve objectives within a constrained budget of $1.5 million to fund Indigenous Protected Areas and stewardship agreements in the catchment. This figure was based on the non-spatial financial estimate of $1 million required for stewardship agreements across pastoral properties in the catchment [18] and a pro-rated estimate of $0.5 million for Indigenous Protected Areas over about 1.5 million ha of Indigenous land. To ensure that scenarios 1 and 2 were directly comparable, we selected a budget for scenario 1 equal to the average area selected in scenario 2 under the constrained budget of $1.5 million (740,000 ha). Similarly, for comparability of scenarios 3 and 4, we selected a budget for scenario 3 equal to the average area selected in scenario 4 under the constrained budget of $1.5 million (620,000 ha).

In scenarios 3 and 4 we include the estimated probability of participation in the stewardship program in the optimization problem to demonstrate how these data might be used in spatial planning. We wanted to select properties with the highest probability of participation while still meeting our objectives within a constrained budget. To do this we included the estimated probability of participation as a conservation feature for each pastoral property and set a catchment-wide objective of 15% of the total probability (which is computationally similar to the approach used by other studies) [11]. The 15% objective was selected to reflect the non-spatial findings of Adams et al. [18] that a $1 million budget would be sufficient to support participation of the most cost-efficient properties (i.e. the largest 15% of properties). Importantly, this approach also allowed probability of participation to be separated from stewardship costs in the software analyses.

We ran Marxan with Zones with 100 runs for each scenario and recorded best solutions and selection frequency for each scenario. For the best solution in each scenario we summarized the total cost and area as well as the cost and area for the stewardship zone (a subset of the areas in the solution for each scenario, Table 2). For properties selected for stewardship we also calculated the average and median property size, percentage of properties selected for stewardship (out of 535 properties), average probability of participation of properties selected, and percentage of total probability. To isolate the effects of including variable costs, we compared scenarios 1 and 2 and scenarios 3 and 4, respectively. To isolate the effects of including probability of participation, we compared scenarios 2 and 4. Scenarios 1 and 3 were not directly comparable because their area budgets were different, having been calibrated, respectively, from scenarios 2 and 4. Lastly, we compared scenarios 3 and 4 to examine the combined effects of including variable costs and probability of participation.

## Results

The final conditional mixed-effects logit model for the choice experiment (Table 1) included the two significant landholder-specific variables - *ln(property size, ha)* and *conservation flag* (p<0.001) - in addition to the two design variables being investigated (configuration and payment). The coefficient for property size was negative, indicating that owners of larger properties were less likely to participate. The coefficient for conservation flag was positive, indicating that owners already engaged in conservation management were more likely to participate. The coefficients for configuration levels were negative, and increasingly so with the extent of change in required proportion of property to be managed for conservation. Accordingly, configuration 3, requiring landholders to change from no patches to one large continuous patch set aside for conservation, had the largest negative coefficient. This trend was similar for both covenants and management agreements. However, the coefficients for covenant configurations were more strongly negative, indicating that landholders were less likely to select a covenant than a management agreement. The coefficient for payment level was positive, indicating that probability of participation increased with payment amount.

For our three payment scenarios, in which payment levels were varied but configuration was held constant, the predicted probabilities of participation in stewardship arrangements increased from 42% to 64% as payment levels increased (Table 3). Respondents always preferred conservation management agreements to covenants. However, the payment premium for covenants substantially increased the probability of participating through a covenant (29% for 150% payment, 18% for 100% payment, Table 3). The design of our choice experiment, lacking an alternative for ‘opting-out’ of negotiations without selling, probably produced absolute probabilities of participating that were higher than an alternative survey design with an ‘opt-out’ choice. However, we expect that the relative probabilities between payment levels and stewardship arrangements reliably indicate the preferences of landholders in the Daly. In fact, the preference of management agreements over covenants was supported by qualitative results from in-person interviews and unsolicited comments provided in survey responses. In addition, if the design of the stewardship program in the Northern Territory comes to reflect the constraints of the Queensland program, coupled with the Delbessie Agreement, then our probabilities will be directly applicable.

**Table 3:** Estimated probabilities of participation for three payment scenarios.

	Payment scenarios		
	50% CC,	100% CC,	150% CC,
	50% CMA	100% CMA	100% CMA
Conservation Covenant (CC)	0.13	0.18	0.29
Conservation Management Agreement (CMA)	0.29	0.40	0.35
Stewardship arrangement (CC + CMA)	0.42	0.58	0.64
Sell Property	0.58	0.42	0.36

CC indicates conservation covenant; CMA indicates conservation management agreement.

In all scenarios, there was approximately 0.5 million ha in existing national parks and Indigenous Protected Areas and an additional 1.5 million ha in buffer areas (the ‘never clear’ zone). Eighty-nine of the 110 objectives were fully achieved in these buffers and protected areas.

For the annual budget of $1.5 million to support management of Indigenous Protected Areas and stewardship agreements, not all conservation objectives could be met (number of missed objectives ranged from 8 to 11 across scenarios, Table 4). In all cases, the 100% targets for the five sites of conservation significance could not be met. Other shortfalls were for rarer vegetation types.

**Table 4:** Summary results from Marxan with Zones for the four scenarios, including number of objectives met, total cost, total cost of stewardship agreements, total area, total area selected for stewardship, average and median property size, percentage of properties selected for stewardship, average probability of participation of selected properties (average probability across all properties is 32.7% with a range from 0.9% to 78.7%), and percentage of total probability of participation in selected properties (objective was 15% for scenarios 3 and 4).

	Objectivesmet(of 110)	Total cost($)	Total Stewardshipcost ($)	Totalarea(ha)	Total area – Stewardship(ha)	Averagepropertysize (ha)	Medianpropertysize (ha)	Percentage ofpropertiesselected forstewardship	AverageProbability	Percentage oftotal probability
Scenario1 - Uniformcost	103	1,962,437	1,125,664	740,000	371,899	11,274	392	45.7%	22.2%	23.4%
Scenario2 - Variablecost	101	1,500,000	623,172	740,000	389,701	18,610	3,717	27.5%	12.6%	2.8%
Scenario3 - Uniformcost + PoP*	103	1,650,119	1,019,919	620,000	280,089	11,794	481	43.6%	21.6%	20.0%
Scenario4 - Variablecost + PoP*	99	1,500,000	822,245	620,000	301,225	12,778	579	40.3%	20.2%	15.0%

*Probability of participation.

Including variable costs reduced the number of properties engaged in stewardship agreements by selecting larger properties, a consequence of strong economies of scale for stewardship costs (compare scenarios 1 and 2 and scenarios 3 and 4, respectively, Table 4). Including variable costs also lowered the overall probability of participation across selected properties because landholders on larger properties were less likely to participate (Table 4). The effects of considering variable costs, in terms of number and size of properties, were more dramatic when probability of participation was not considered (compare scenarios 1 and 2, Table 4). With probability of participation also included, these effects of variable costs were tempered (compare scenarios 3 and 4, Table 4) by the inverse relationship between cost and probability of participation, below.

Including variable costs and probability of participation (scenario 4) shifted spatial selections to smaller properties with higher probability compared to using variable costs only (scenario 2), and this increased the number of missed objectives marginally to 11, compared to 9 in scenario 2. In relation to scenario 2, the average probability of participation of selected properties in scenario 4 almost doubled, the percentage of total probability in selected properties increased five-fold, and the median property size dropped from 3,717 ha to 579 ha (Table 4).

Compared to probability of participation with uniform costs (scenario 3), including both probability of participation and variable costs (scenario 4) decreased overall probability of participation. This was because including variable costs slightly increased the average size of properties, due to economies of scale, but also reduced the average probability of participation, due to the negative relationship between property size and probability of participation (Table 1). This reflects a tension between two key considerations in the Daly: cost-effectiveness requires that larger properties should be targeted for stewardship, but overall probability of participation is thereby lowered.

## Discussion

Choice modelling has been applied to management of protected areas or design of conservation incentives [22], [23] but, to our knowledge, it has not previously been combined with conservation planning for optimal spatial design of a stewardship program. Our choice analysis provides several insights for designing and implementing a stewardship program in the Northern Territory. We estimated that a large percentage of landholders – between 42% to 64%, depending on payment levels - would be willing to participate in stewardship agreements (i.e. a covenant or a management agreement). We found that landholders were financially motivated in their preferences between conservation management agreements and conservation covenants. All else being equal, landholders preferred management agreements, reflecting their reported concerns over the title implications of covenants and potential negative effects on sale values. This is consistent with previous reports of respondents’ concerns over agreements impinging upon their rights to use and manage land [20] and previous findings that shorter or less restrictive management agreements are preferred [24]. However, this preference for management agreements in the Daly can apparently be weakened with a payment premium for covenants. Covenants have benefits for the Government. The first is the security of permanent titling [7]. Second, titling allows covenants to be classified as IUCN-recognized protected areas (Class VI in the case of Nature Refuges, however private protected areas may qualify for all classes) [38] so that covenants then contribute to national conservation goals such as the 2020 17% target under the Convention on Biological Diversity [39].

The stewardship payment model developed by Adams et al. [18] found strong economies of scale with the largest properties being the most cost-efficient. However, in our choice model, the negative coefficient associated with *ln(property size, ha)* indicated that the most cost-efficient properties were also the least likely to participate. This finding could reflect the tendency for larger properties to be more likely associated with production land uses, with production landholders more concerned about lost income from stewardship agreements than non-production landholders on smaller properties [20]. Our spatial zonings supported the findings of Adams et al. [18] that including variable stewardship costs to select the most cost-efficient implementation of the stewardship program resulted in engaging with larger properties. However, our analyses here also demonstrated that the budget level of $1.5 million per annum was insufficient for all conservation objectives to be met. Furthermore, if the stewardship program were implemented as a closed-bid auction, probably even fewer conservation objectives would be met because landholders on the most cost-efficient properties would be less likely to submit bids. Rather, the more willing participants would be more likely to have smaller properties that are more costly to manage per ha, and a larger budget would therefore be needed to meet conservation objectives while engaging these landholders. Therefore, our analysis indicates that, to meet the kinds of conservation objectives used here, the design of the stewardship program would need to encourage participation of large properties by addressing their managers’ specific concerns about enrolling [8], [20] or involve a larger budget than $1.5 million to engage small properties. Alternatively the Government might fund an outreach campaign prior to starting the stewardship program to increase the probability of larger properties participating.

Our choice experiment sought to mimic a key characteristic of the Delbessie Agreement by explicitly not offering landholders a choice to ‘opt-out’. We believe, however, that our experimental design would not have exaggerated one of our key conclusions: that property size was inversely related to probability of participation, creating a tension between selecting properties that are cost-efficient and selecting properties with landholders who are willing to participate. Our design would have exaggerated the negative association between property size and probability of participating only if probability of selling and property size were positively related, that is, if owners of larger properties were more likely to sell than those of smaller properties. In that case, having the option to sell rather than to engage in stewardship would be more appealing to owners of larger properties. However, we found that property size and number of years of ownership, admittedly an imprecise proxy for propensity to sell, were uncorrelated. We conclude that it is unlikely that an alternative experimental design would have changed our observed negative association between property size and probability of participation.

The number of studies considering variable conservation costs has increased recently, demonstrating the benefits associated with incorporating costs into priority setting [40]. Recent advances have included more sophisticated dynamics such as land-market feedbacks [41], [42]. However, studies of variable costs typically assume uniform availability of land. Specifically, they fail to consider that some landholders will be more or less willing to engage in conservation management, whether by selling their land or participating in stewardship programs. Progress on incorporating costs parallels advances in integrating other social considerations in systematic planning, such as measures of willingness or social indicators of feasibility. These studies have demonstrated ways of making plans more readily implemented [43], [44]. Variation in landholders’ willingness to participate, similar to our approach here, has been considered in two other spatial prioritizations [11], [45], but those studies included costs (unrealistically) as uniform, average sales prices across properties. To our knowledge, no previous study has included both spatially variable costs and spatial variation in willingness to participate in spatial optimization.

By selecting areas with spatially variable data on both costs and willingness to participate, our study demonstrated that, with a constrained budget, spatially variable costs can be more important than willingness in determining conservation priorities. This is likely to be the case more generally, where economies of scale apply to costs such as those of acquisition and management [46]–[48]. This result provides an important insight into the potential interactions between the spatial distribution of conservation features, costs of conservation, and willingness of landholders to engage in conservation. These interactions will be important to consider for future studies concerned with opportunities for and constraints on implementation. Our analyses highlight important design and policy issues associated with implementing a stewardship program in the Northern Territory and other parts of the world. If planners understand the spatial drivers of both costs and probability of participation, then trade-offs can be addressed pro-actively with engagement strategies or arguments for adequate budgets.

## Supporting Information

Survey S1 **Survey and cover letter designed for and used in this study.**
(PDF)
