# Supplementary material for: Estimating Landholders’ Probability of Participating in a Stewardship Program, and the Implications for Spatial Conservation Priorities
Source: PLoS One. 2014 Jun 3;9(6):e97941. doi: 10.1371/journal.pone.0097941 (PMC4043528; doi:10.1371/journal.pone.0097941)
Supplement: Survey S1 — Survey and cover letter designed for and used in this study. (PDF) [file pone.0097941.s001.pdf]

Survey S1

The project seeks to understand your preferences for dealing with conservation matters on your land and the financial, biological and political reasons for this. As such, we are trying to reach all landholders in the Daly Catchment (extending from the coast, south-west of Darwin, about 250 km inland) with land parcels of at least 10ha.

As you are aware, the clearing moratorium in the Daly area as well as the land clearing cap has maintained much of the native vegetation. However, changing climate and fire regimes, as well as increased invasive weeds and feral animals pose a large threat to native vegetation and are causing losses of native vegetation and animals. In order to maintain healthy native systems your aid is necessary to control invasive species and continue healthy fire regimes.

We hope that our survey will help answer important questions such as

- What would best reflect your preferences for conservation – payment schemes to support conservation management agreements or to establish conservation covenants?
- What support you would need to be able to engage in the preferred conservation actions?

We have attached the questionnaire to this letter. We would be very grateful if someone in the household could fill it in (only one response is needed), RETAIN THIS COVER LETTER (so that your response remains confidential) and then mail it back to us in the enclosed reply-paid envelope. As noted in our earlier letter, all information which we collect will be kept **strictly confidential**. No information will be attributed to any single person or household, and results will only be released in aggregate form. Responses to the survey will be stored separately from the names and addresses of households, so that no link can be made between them.

Should you have any queries about the project or if you are interested in seeing the results of our survey please do not hesitate to contact me.

On behalf of the JCU research team, thank you in advance for your assistance.

Yours sincerely,

Vanessa Adams

CURRENT LAND USE AND MANAGEMENT

First, we would like to find out about how you use your land and what your current land use management practices are.

1. Are you the owner of the property or the manager?
☐ Owner ☐ Other *(please specify)*......................

2. If you are the owner or manager of multiple properties, how many properties are you referring to in your responses for this survey *(please indicate the number of properties, and if available something that would identify those properties such as the address or the 'laiskey' of the properties)*.
Number (and address or 'laiskey')..

3. Are you willing to answer a survey individually for each of the properties that you indicated you manage in question 2?
☐ I would like a separate survey to complete for each property, please send me additional surveys.
☐ I would like to answer the survey in aggregate for all my properties, do not send me additional surveys.

If you have indicated that you will answer a survey for each property, please indicate the address or 'laiskey' of the property you refer to for the rest of the survey ....

4. How long have you (or your family) been running this property? *Please indicate years.*
Years.....................................

5. What is your age?
☐ <30 ☐ 30-39 ☐ 40-49 ☐ 50-59 ☐ 60-69 ☐ >70

6. What is your gender?
☐ Male ☐ Female

7. What tenure is the property under?
☐ freehold ☐ leasehold ☐ other *(please specify)*.......................

8. How many people are working on the property full time and part time (including contract)? How many are family members (including yourself)?

In total: Full time…… Part time…… Part time hrs/wk *(Average hrs/week per part time staff)* ……

Family: Full time…… Part time…… Part time hrs/wk *(Average hrs/week per part time staff)* ……

9. In general, would you like to see your land maintained:
☐ In its current state ☐ In a more natural state ☐ In a more developed state

10. In general would you like to see the Daly catchment as a whole managed at:
☐ In its current state ☐ In a more natural state ☐ In a more developed state

11. What is the approximate size of your property? *Please indicate the area measure you use.*
Size..hectares acres square km square miles

12. Approximately how much of your property is used for grazing? *Please indicate the area measure you use.*

Grazing area.............................. hectares acres square km square miles

And of the land used for grazing approximately how much occurs on land that is (use the same area measure used above):

Bush and unimproved country................

Cleared land with natural pasture.......................

Cleared land with introduced pasture or fodder crops.................

13. Approximately how much of your property is used for intensive agricultural use (such as dry land, irrigated and fallow crops or other horticultural uses such as plantations)? *Please indicate the area measure you use.*

Area.. hectares acres square km square miles

And of the land used for agriculture approximately how much area is used for each of the following types of intensive use (use the same area measure used above):

Dry land Crops................

Irrigated Crops................

Follow Crops..................

Forest Plantation.............

14. Approximately how much of your property is used for tourism? *Please indicate the area measure you use.*

Area.. hectares acres square km square miles

And of the land used for tourism how much area is used for each of the following types of intensive use (use the same area measure used above):

Native vegetation............

Land unsuitable for any agricultural use............

Land already cleared and used................

15. Approximately how much of your property is used for other uses (e.g. residential or other industries not already mentioned)? *Please indicate the area measure you use.*

Area.. hectares acres square km square miles

Please list how land is used...... ....

16. Do any of the following pose threats to your land? If not, please indicate 0 for "Not Present". If present please indicate the prominence of each threat with 1 being low threats and 5 being a large threat to the entire property:

	Not Present	Threatens only some of property		Threatens about half of the property		Threatens entire property
	0	1	2	3	4	5
Uncontrolled fires	☐	☐	☐	☐	☐	☐
Declared Weeds* such as *Mimosa pigra*	☐	☐	☐	☐	☐	☐
Other weeds	☐	☐	☐	☐	☐	☐
Feral animals (pigs, buffalo etc.)	☐	☐	☐	☐	☐	☐
Unauthorised Access (hunting etc.)	☐	☐	☐	☐	☐	☐

*the Northern Territory declared weed list is available at: http://www.nt.gov.au/nreta/natres/weeds/legislation.html

We know that there are laws which require you to undertake certain actions to manage your property. We are interested in asking you more about how much it costs you and how long it takes you to undertake these actions to meet these obligations. Examples of actions you may be required to take to maintain your land are:

Minimum requirements	• Control weeds<br>• Control feral animals<br>• Control erosion<br>• Minimise fire hazards

We would also like to know about any extra environmental actions that you take that go above and beyond your legislative obligations. For example this could be to prevent and mitigate threats to natural values before they are actually being damaged. Actions that may be beyond requirements are, for example:

Actions beyond minimum requirements	• Fencing to protect rainforest patches and watercourses<br>• Revegetation of threatened plants<br>• Weed control for non declared weed species<br>• Strategic and extensive feral animal control efforts<br>• Patch burning in bush areas to minimise risk of large fires and promote regeneration of native species

17. How would you describe the time/effort required to meet the minimum required land management obligations?

- ☐ Requires little extra work
- ☐ Is a moderate amount of hours and dollars
- ☐ Is too demanding, both in hours and dollars, to meet standards

18. On average how many days a month do you dedicate to managing your land to meet these required management obligations?

☐ <1 ☐ 1-2 ☐ 3-7 ☐ >7

19. On average how much do you spend in a year on managing your land to meet these management obligations? Please answer in dollars for the itemized list below:

Item	Amount ($) and any details you can provide
Supplies (regular operation costs such as fuel, pesticides and fencing materials such as timber and wire)	
Labor (wages and salaries that reflect your time, your employee’s time and hired services such as culling of feral animals)	
Other cost (please specify, e.g. replacement of worn-out equipment and investment in new facilities)	

20. How often do you undertake the following to manage your land:

	Frequency				
	Never	Weekly	Monthly	Quarterly	Annually
Back burn away from areas that are fire sensitive	☐	☐	☐	☐	☐
Control weedy grasses (such as Mission Grass) that increase fuel loads	☐	☐	☐	☐	☐
Control other type of weeds with fire	☐	☐	☐	☐	☐
"Cool burn" early in dry season or after first wet storm to break up fuel layer (indicate whether you undertake (annually) or not (never))	☐				☐
Control weeds mechanically	☐	☐	☐	☐	☐
Control weeds with chemicals	☐	☐	☐	☐	☐
Use integrated or biological pest control to remove weeds	☐	☐	☐	☐	☐
Cull feral animals	☐	☐	☐	☐	☐

21. We understand that a common method for protecting land against feral animals is to put up fencing. Please indicate the percentage of your land that you have fenced off to protect against feral animals: ....

22. If there are actions you take to manage your land beyond legislative obligations please list them and the approximate cost of undertaking such actions:

Item	Amount ($) and any details you can provide including breakdown of labor, operational costs such as fuel or other supplies, maintenance of existing facilities or investment in new facilities
For example, you may have fenced off rainforest patches on your property	S$ in fencing supplies<br>T$ in paid labor to construct fences<br>Y hrs supervising and aiding in construction of fences<br>Z hrs annually in fence maintenance

23. Aside from areas that you may be required to leave uncleared such as riparian strips along creeks or wetlands, do you have native vegetation or other natural features that you are managing, including required management actions and environmental actions beyond the minimum required? What spatial configuration below best describes the placement of these areas on your land? *Please refer to the diagrams and indicate what spatial configuration is most representative. Note grey indicates your property and black indicates areas being managed. If you feel that A – D are not applicable please use E to draw the configuration that applies to your property.*

- ☐ A) None
- ☐ B) Limited and patchy
- ☐ C) Extensive and patchy
- ☐ D) Extensive and continuous
- ☐ E) None of these are applicable, I will draw my own in

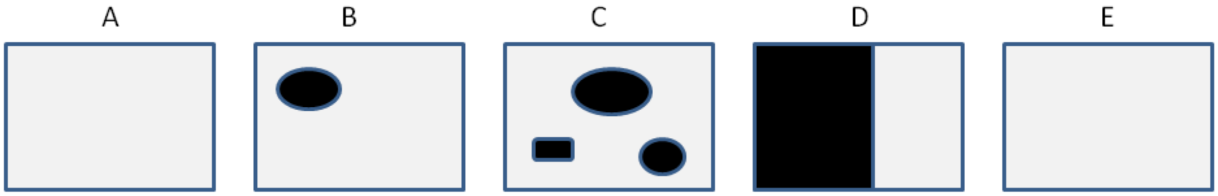

Now, we would like to ask about your familiarity with different land management options. For your reference, conservation covenants and conservation management agreements are described below:

A **conservation covenant** is a legally binding agreement (similar to a power line easement) that is held with the land title. The covenant is voluntarily entered into, and includes land management restrictions that are aimed to protect special natural values on the property (such as a population of threatened species). Land uses such as grazing may be compatible with the covenant depending on the requirements of the special natural values the covenant is to protect. Covenants can cover either all or a specific portion of a land title. There are no native title implications as the agreement is not a subdivision and tenure does not change.

A **conservation management agreement (CMA)** is a contract or binding agreement regarding the use and management of a portion of land that has special natural values. A CMA specifies conservation actions and or outcomes that you agree. *A CMA differs from a conservation covenant in that it is not placed against the land title and thus does not bind future owners of the property if it is to change hands.*

24. Please indicate your familiarity with each of the management options, from "not at all" to "very":

	Not familiar				Very familiar
	1	2	3	4	5
Current land management obligations	☐	☐	☐	☐	☐
Conservation Management Agreement	☐	☐	☐	☐	☐
Conservation Covenant	☐	☐	☐	☐	☐

We would like to ask you a little bit more about what capacity, infrastructure, or financial support you need to undertake increased conservation management on your land.

If government funding was available to run conservation schemes the payments provided to you, under a legal agreement, to support specific conservation actions may be in many forms such as providing:

- People (e.g. **Indigenous Rangers or Conservation Managers)** that will undertake management actions such as controlled burning on your land
- **Supplies** such as chemicals for weed control or fencing materials
- Public recognition (such as **Conservation Certification)** for your property
- **Offsets** such as increased clearing rights
- **Financial payments**

25. Please indicate your interest in each of the management options, from "would not consider" to "very":

	Would not consider 0	1	2	3	4	Would be very interested 5
Indigenous Rangers or Conservation Managers	☐	☐	☐	☐	☐	☐
Supplies	☐	☐	☐	☐	☐	☐
Conservation Certification	☐	☐	☐	☐	☐	☐
Offsets	☐	☐	☐	☐	☐	☐
Financial Support	☐	☐	☐	☐	☐	☐

Choice Sets

Finally, we would like to find out more about your preferences with respect to conservation arrangements and compensation for undertaking conservation on your property.

We will do this by presenting you with 10 different HYPOTHETICAL situations. In each situation, you will be told what the HYPOTETICAL aim/goal of the government is - and will be shown a picture of how this would affect your HYPOTHETICAL property. You will then be given a set of options, namely to:

a) agree to change your property, using a CONSERVATION CONVENANT – and receiving the compensation specified in the hypothetical example

b) agree to change your property, using a CONSERVATION MANAGEMENT AGREEMENT – receiving the level of compensation specified in the example; or

c) sell the entire property – at the going market rate.

For each of the 10 different hypothetical situations, we ask you to indicate which of the options (agree to a covenant; negotiate a management agreement; or sell) you would select. *To indicate your choice, simply tick the box next to the option you select. Please make sure to indicate your selection for each of the 10 sets and consider only the options presented in a set to determine your preference.*

Example choice set:

Imagine that the government would like you to set aside two more 'patches' of land on your property for conservation purposes. This would change the configuration of your land

From:	***To:***
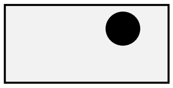 **where there is one small patch set aside for conservation**	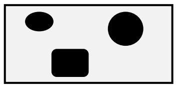 **where there are several patches set aside for conservation**

Would you choose to	**Payment (as a % of Total Costs)**	**Choice** ☑
Accept a **Conservation covenant**, that would require you to pay for the survey costs, and then spend 1-2 days per month 'managing' the extra conservation areas	*and* receive compensation for **50%** of all costs	☑
Or Accept a **Conservation management agreement**, that would require you to purchase some extra supplies (e.g. fencing) and labour (to put the fences in) and that would require you to spend an extra 1-2 days per month 'managing' the areas	*and* receive compensation for **50%** of all costs	☐
Or **Sell your entire property at market value**	Market value	☐

You would consider the options presented here and select ONE of the three options by ticking the appropriate box. If, you prefer the conservation covenant, then you would tick the first box.

1. Imagine that the government would like you to set aside several 'patches' of land on your property for conservation purposes. This would change the configuration of your land

From:	***To:***
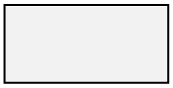	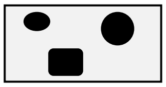
**where there are no patches set aside for conservation**	**where there are several patches set aside for conservation**

Would you choose to	**Payment (as a % of Total Costs)**	**Choice**
Accept a **Conservation covenant**, that would require you to pay for the survey costs, and then spend 1-2 days per month 'managing' the extra conservation areas	*and* receive compensation for **150%** of all costs	☐
Or Accept a **Conservation management agreement**, that would require you to purchase some extra supplies (e.g. fencing) and labour (to put the fences in) and that would require you to spend an extra 1-2 days per month 'managing' the areas	*and* receive compensation for **150%** of all costs	☐
Or **Sell your entire property at market value**	Market value	☐

2. Imagine that the government would like you to set aside one 'patch' of land on your property for conservation purposes. This would change the configuration of your land

From:	***To:***
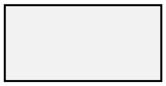	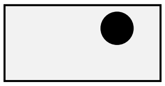
**where there are no patches set aside for conservation**	**where there is one small patch set aside for conservation**

Would you choose to	**Payment (as a % of Total Costs)**	**Choice**
Accept a **Conservation covenant**, that would require you to pay for the survey costs, and then spend 1-2 days per month 'managing' the extra conservation areas	*and* receive compensation for **0%** of all costs	☐
Or Accept a **Conservation management agreement**, that would require you to purchase some extra supplies (e.g. fencing) and labour (to put the fences in) and that would require you to spend an extra 1-2 days per month 'managing' the areas	*and* receive compensation for **50%** of all costs	☐
Or **Sell your entire property at market value**	Market value	☐

3. Imagine that the government would like you to set aside two more 'patches' of land on your property for conservation purposes. This would change the configuration of your land

From:	***To:***
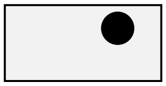	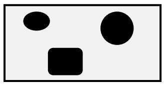
**where there is one small patch set aside for conservation**	**where there are several patches set aside for conservation**

Would you choose to	**Payment (as a % of Total Costs)**	**Choice**
Accept a **Conservation covenant**, that would require you to pay for the survey costs, and then spend 1-2 days per month 'managing' the extra conservation areas	*and* receive compensation for **100%** of all costs	☐
Or Accept a **Conservation management agreement**, that would require you to purchase some extra supplies (e.g. fencing) and labour (to put the fences in) and that would require you to spend an extra 1-2 days per month 'managing' the areas	*and* receive compensation for **150%** of all costs	☐
Or **Sell your entire property at market value**	Market value	☐

4. Imagine that the government would like you to set aside several 'patches' of land on your property for conservation purposes. This would change the configuration of your land

From:	***To:***
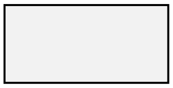	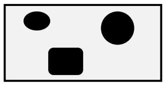
**where there are no patches set aside for conservation**	**where there are several patches set aside for conservation**

Would you choose to	**Payment (as a % of Total Costs)**	**Choice**
Accept a **Conservation covenant**, that would require you to pay for the survey costs, and then spend 1-2 days per month 'managing' the extra conservation areas	*and* receive compensation for **0%** of all costs	☐
Or Accept a **Conservation management agreement**, that would require you to purchase some extra supplies (e.g. fencing) and labour (to put the fences in) and that would require you to spend an extra 1-2 days per month 'managing' the areas	*and* receive compensation for **50%** of all costs	☐
Or **Sell your entire property at market value**	Market value	☐

5. Imagine that the government would like you to set aside several 'patches' of land on your property for conservation purposes. This would change the configuration of your land

From:	***To:***
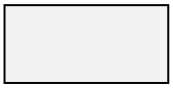 **where there are no patches set aside for conservation**	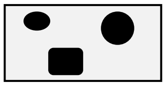 **where there are several patches set aside for conservation**

Would you choose to	**Payment (as a % of Total Costs)**	**Choice**
Accept a **Conservation covenant**, that would require you to pay for the survey costs, and then spend 1-2 days per month 'managing' the extra conservation areas	*and* receive compensation for **50%** of all costs	☐
Or Accept a **Conservation management agreement**, that would require you to purchase some extra supplies (e.g. fencing) and labour (to put the fences in) and that would require you to spend an extra 1-2 days per month 'managing' the areas	*and* receive compensation for **50%** of all costs	☐
Or **Sell your entire property at market value**	Market value	☐

6. Imagine that the government would like you to set aside a continuous section of land on your property for conservation purposes. This would change the configuration of your land

From:	***To:***
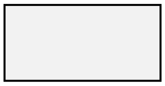 **where there are no patches set aside for conservation**	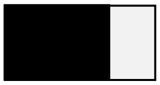 **where there is one continuous area set aside for conservation**

Would you choose to	**Payment (as a % of Total Costs)**	**Choice**
Accept a **Conservation covenant**, that would require you to pay for the survey costs, and then spend 1-2 days per month 'managing' the extra conservation areas	*and* receive compensation for **150%** of all costs	☐
or Accept a **Conservation management agreement**, that would require you to purchase some extra supplies (e.g. fencing) and labour (to put the fences in) and that would require you to spend an extra 1-2 days per month 'managing' the areas	*and* receive compensation for **50%** of all costs	☐
or **Sell your entire property at market value**	Market value	☐

7. Imagine that the government would like you to set aside a continuous section of land on your property for conservation purposes. This would change the configuration of your land

From: 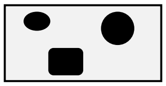

where there are several patches set aside for conservation

To: 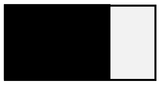

where there is one continuous area set aside for conservation

Would you choose to	**Payment (as a % of Total Costs)**	**Choice**
Accept a **Conservation covenant**, that would require you to pay for the survey costs, and then spend 1-2 days per month 'managing' the extra conservation areas	*and* receive compensation for **100%** of all costs	☐
or<br>Accept a **Conservation management agreement**, that would require you to purchase some extra supplies (e.g. fencing) and labour (to put the fences in) and that would require you to spend an extra 1-2 days per month 'managing' the areas	*and* receive compensation for **0%** of all costs	☐
or<br>**Sell your entire property at market value**	Market value	☐

8. Imagine that the government would like you to set aside a continuous section of land on your property for conservation purposes. This would change the configuration of your land

From: 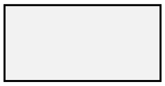

where there are no patches set aside for conservation

To: 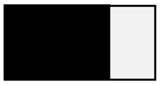

where there is one continuous area set aside for conservation

Would you choose to	**Payment (as a % of Total Costs)**	**Choice**
Accept a **Conservation covenant**, that would require you to pay for the survey costs, and then spend 1-2 days per month 'managing' the extra conservation areas	*and* receive compensation for **0%** of all costs	☐
or<br>Accept a **Conservation management agreement**, that would require you to purchase some extra supplies (e.g. fencing) and labour (to put the fences in) and that would require you to spend an extra 1-2 days per month 'managing' the areas	*and* receive compensation for **150%** of all costs	☐
or<br>**Sell your entire property at market value**	Market value	☐

9. Imagine that the government would like you to set aside two more 'patches' of land on your property for conservation purposes. This would change the configuration of your land

From:	***To:***
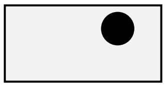	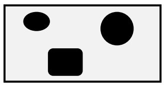
**where there is one small patch set aside for conservation**	**where there are several patches set aside for conservation**

Would you choose to	**Payment (as a % of Total Costs)**	**Choice**
Accept a **Conservation covenant**, that would require you to pay for the survey costs, and then spend 1-2 days per month 'managing' the extra conservation areas	*and* receive compensation for **50%** of all costs	☐
or Accept a **Conservation management agreement**, that would require you to purchase some extra supplies (e.g. fencing) and labour (to put the fences in) and that would require you to spend an extra 1-2 days per month 'managing' the areas	*and* receive compensation for **100%** of all costs	☐
or **Sell your entire property at market value**	Market value	☐

10. Imagine that the government would like you to set aside one 'patch' of land on your property for conservation purposes. This would change the configuration of your land

From:	***To:***
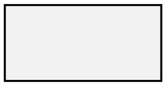	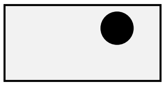
**where there are no patches set aside for conservation**	**where there is one small patch set aside for conservation**

Would you choose to	**Payment (as a % of Total Costs)**	**Choice**
Accept a **Conservation covenant**, that would require you to pay for the survey costs, and then spend 1-2 days per month 'managing' the extra conservation areas	*and* receive compensation for **50%** of all costs	☐
or Accept a **Conservation management agreement**, that would require you to purchase some extra supplies (e.g. fencing) and labour (to put the fences in) and that would require you to spend an extra 1-2 days per month 'managing' the areas	*and* receive compensation for **0%** of all costs	☐
or **Sell your entire property at market value**	Market value	☐

GENERAL HOUSEHOLD INFORMATION

Lastly, we would like to know a little bit more about your household. This information will be kept strictly confidential and is used to ensure that we have collected information from a wide variety of households.

Please tick appropriate box for each question.

1. What is the total, combined, annual (taxable) income of ALL the people who 'normally' live in your house?

☐ <$20,000 AUS Dollars
☐ $20,000-$40,000 AUS Dollars
☐ $40,000-$60,000 AUS Dollars
☐ $60,000-$80,000 AUS Dollars
☐ $80,000-$100,000 AUS Dollars
☐ $100,000-$120,000 AUS Dollars
☐ $120,000-$140,000 AUS Dollars
☐ $140,000-$160,000 AUS Dollars
☐ > $160,000 AUS Dollars

2. Please indicate the percentage of your income that you derive from enterprises on your property and how much of your income is from work off of the land:

On land enterprises: ………
Off farm income: ………

3. What enterprises do you have on your property? Please indicate the percentage of your on farm income that you derive from these enterprises. *Please tick appropriate box(es)*

	Percent Income				
	0%	25%	50%	75%	100%
Cattle Grazing on:					
Bush and Unimproved Country	☐	☐	☐	☐	☐
Cleared Land with natural pasture	☐	☐	☐	☐	☐
Cleared Land with Introduced Pasture or Fodder Crops	☐	☐	☐	☐	☐
Intensive Use for:					
Dry land Crops	☐	☐	☐	☐	☐
Irrigated Crops	☐	☐	☐	☐	☐
Fallow Crops	☐	☐	☐	☐	☐
Forest Plantation	☐	☐	☐	☐	☐
Tourism					
Property Stay	☐	☐	☐	☐	☐
Access to Tour Provider	☐	☐	☐	☐	☐
Other (specify: _____________)	☐	☐	☐	☐	☐
Other (Please Specify)					
	☐	☐	☐	☐	☐
	☐	☐	☐	☐	☐
